# The Diverse Functions of the Ubiquitous Fcγ Receptors and Their Unique Constituent, FcRγ Subunit

**DOI:** 10.3390/pathogens9020140

**Published:** 2020-02-20

**Authors:** Thamer A. Hamdan, Philipp A. Lang, Karl S. Lang

**Affiliations:** 1 Institute of Immunology, Medical Faculty, University of Duisburg-Essen, Hufelandstraße 55, 45147 Essen, Germany; 2 Department of Molecular Medicine II, Medical Faculty, Heinrich Heine University, Universitätsstrasse 1, 40225 Düsseldorf, Germany

**Keywords:** FcγRs, FcRγ, NCR1, CD8^+^ T cells, chronic viral infections

## Abstract

Fc gamma receptors (FcγRs) are widely expressed on a variety of immune cells and play a myriad of regulatory roles in the immune system because of their structural diversity. Apart from their indispensable role in specific binding to the Fc portion of antibody subsets, FcγRs manifest diverse biological functions upon binding to their putative ligands. Examples of such manifestation include phagocytosis, presentation of antigens, mediation of antibody-dependent cellular cytotoxicity, anaphylactic reactions, and the promotion of apoptosis of T cells and natural killer cells. Functionally, the equilibrium between activating and inhibiting FcγR maintains the balance between afferent and efferent immunity. The γ subunit of the immunoglobulin Fc receptor (FcRγ) is a key component of discrete immune receptors and Fc receptors including the FcγR family. Furthermore, FcγRs exert a key role in terms of crosslinking the innate and adaptive workhorses of immunity. Ablation of one of these receptors might positively or negatively influence the immune response. Very recently, we discovered that FcRγ derived from natural cytotoxicity triggering receptor 1 (NCR1) curtails CD8^+^ T cell expansion and thereby turns an acute viral infection into a chronic one. Such a finding opens a new avenue for targeting the FcγRs as one of the therapeutic regimens to boost the immune response. This review highlights the structural heterogeneity and functional diversity of the ubiquitous FcγRs along with their featured subunit, FcRγ.

## 1. Introduction

The receptors of the fragment crystalizable (Fc) portion of immunoglobulin (Fc receptors, FcRs) are ubiquitously found on a variety of cell types of the immune system with versatile functions. They belong to the large immunoglobulin (Ig) superfamily, and are type I transmembrane glycoproteins (the carboxyl terminus of the polypeptide chain is located in the cytosol, whereas the amino terminus is exposed to the extracellular space) [1,2]. 

On the basis of the type of antibody they recognize, FcRs are mainly classified as FcαR, which binds to IgA; FcδR, which binds to IgD; FcεR, which binds to IgE; FcγR and the neonatal Fc receptor, which bind to IgG; and FcµR, which binds to IgM [3,4]. FcγRs perform a wide range of functions impacting afferent and efferent immunity. In addition to their essential role in specific binding to the Fc portion of antibody subsets, their binding to immune complexes on dendritic cells (DCs) and macrophages (Mφ) leads to phagocytosis and the presentation of antigenic peptides via major histocompatibility complex (MHC) class I and class II proteins. These proteins are further recognized by T cell subsets, resulting in the activation of these cells and the mediation of their functions accordingly [5,6].

Generally, FcRs are among the immune receptors that recognize antigens indirectly along with B cell receptors (BCRs) and T cell receptors (TCRs); these receptors have activation motifs and signaling pathways in common. FcRs recognize the Fc portion of an antibody rather than antigens directly, forming a complex of membrane-bound receptors for antigens. Several factors dictate the outcomes of FcR-antibody engagement that affect the expression levels of activating and inhibitory FcγRs by virtue of cytokines or alteration in the affinity of antibody-FcγR binding due to differential antibody glycosylation. FcRs prevail in two forms: membrane receptors and soluble molecules, generated by alternative splicing of FcR transcripts or by proteolysis of membrane receptors, and these have distinct roles in B cell proliferation and antibody production [1,7,8]. 

The γ subunit of the immunoglobulin Fc receptor (FcRγ) is a salient homodimeric part of various Fc receptors—namely, the high-affinity receptor for IgE (FcεRI in mice and humans), the high-affinity receptor for IgG (FcγRI or CD64 in mice and humans), the low- to medium-affinity receptor for IgG (FcγRIII or CD16 in mice and FcγRIIIA in humans), and the low-affinity receptor for IgA (FcαRI or CD89 in humans). Moreover, Fcγ associates as a heterodimeric unit with various immunoreceptors, such as NKp46; the platelet collagen receptor glycoprotein VI; the paired immunoglobulin-like receptor A (PIR-A), which is homologous to human CD85; the interleukin (IL)-3 receptor; the osteoclast-associated receptor (OSCAR); signal-regulatory protein β1 (SIRPβ1); triggering receptor expressed on myeloid cells (TREM); and Dectin-1 [1,9,10,11,12]. Collectively, the γ subunit is indispensable not only as a structural part of Fc receptors but also as a pivotal mediator of an array of immune functions, including signal transduction [10]. In this context, FcRγ is also called FcεRIγ because FcRγ was first noticed as the third subunit of FcεRIγ and subsequently as a common gamma chain on FcγRIII (CD16), FcγRI (CD64), and FcαRI (CD89) [12,13,14]. 

NCR1/NKp46 is a natural killer (NK) cell activation marker and is deemed a prototypical example of an FcRγ-associated molecule. More specifically, FcRγ is exquisitely bound to the transmembrane region of NKp46 and plays a prominent role in NK cell activation signaling. Moreover, the transmembrane part of FcRγ is joined by a disulfide bond to CD3ζ, and both of them contain immunoreceptor tyrosine-based activation motifs (ITAMs) in their cytoplasmic tails; these ITAMs initiate signaling downstream to NKp46 [15,16]. Recently, we found that NCR1-associated FcRγ negatively affects the CD8 T cell response both qualitatively and quantitatively in the context of chronic infection with lymphocytic choriomeningitis virus (LCMV); this finding emphasizes the function of FcRγ as a modulator in innate and adaptive immunity [11]. Nevertheless, the intrinsic impact of FcRγ on immune cells other than NK cells during chronic viral infection remains to be investigated. In this review, we recapitulate the structural and functional aspects of FcγR and its unique subunit, FcRγ, which is present in virtually all FcγRs—except for CD32 (FcγRII)—in the immune response. 

## 2. Structure of FcγRs

The conformational states of the Fc domain determine the identity of FcRs; two distinct sets of FcRs have been identified for IgG: the canonical type I FcRs that bind the open conformation of Fc domain—which belong to the Ig receptor superfamily and include the activating receptors FcγRI, FcγRIIa, FcγRIIc, FcγRIIIa, and FcγRIIIb—and the inhibitory receptor FcγRIIb. On the other hand, type II FcRs are represented by the family of C-type lectin receptors, which includes CD209 (DC-SIGN; homologous to SIGN-R1 in mice) and CD23. Along with the Fab portion, the heterogeneity of effector molecules that are engaged by the Fc domain imparts polyclonality to antibodies [17,18].

The high-affinity FcγRIs (FcγRIA, FcγRIB, and FcγRIC) are encoded by a single mouse gene but by three human genes. Moreover, the low-affinity FcγRs (FcγRIIs, such as FcγRIIA, FcγRIIB, and FcγRIIC; and FcγRIIIs, such as FcγRIIIA and FcγRIIIB) are encoded by two mouse genes but by five human genes. FcεRI is a tetramer consisting of one alpha chain, one beta chain, and two gamma chains. These gamma chains are also a shared subunit of other Fc receptors and are encoded by the *fcer1g* gene, which contains five exons and spans four kilobases. This gene evolved from the same ancestor as the zeta chain of T cell receptors; both of these genes are located on the long arm of chromosome 1 (1*q*21–23) [13,19,20,21], as shown in Table 1. 

## 3. FcγR Signaling Pathways

Fc receptors exist as activation receptors and relay their signals via ITAMs, and as inhibitory receptors that transmit their signals via the immunoreceptor tyrosine-based inhibitory motif (ITIM) [22,23]. The activation of FcγRs requires a ligand-binding α-chain adaptor molecule containing ITAMs that is extended in its cytoplasmic domain to activate signaling pathways. Structurally, adaptor proteins differ among immune cells. For example, FcγRIIIA on NK cells is associated with the T cell receptor z-chain [21], yet FcγRIIIA associates with the common γ-chain in monocytes and macrophages. Furthermore, FcεRI and the FcγRIII in basophils and mast cells have an extra β-chain [24]. Upon the crosslinking with immune complexes, the signaling pathway begins with the tyrosine phosphorylation of the ITAMs linked to the γ chain via SRC family kinases (SFKs), such as Fyn and Lyn. These ITAMs form docking sites for SYK family kinases, culminating in the subsequent recruitment of other downstream proteins, such as phosphoinositide 3-kinase (PI3K). These activate downstream kinases and lead to the release of calcium from the endoplasmic reticulum (ER), and of granular content, which includes perforin and granzymes [25,26]. Notwithstanding, the inhibitory FcγRIIB transduces inhibitory signals by phosphorylating the tyrosines present in ITIMs. This inhibition requires ligation between the activating heterologous receptor (e.g., the BCR) and the inhibitory FcR, which is mediated by immune complexes. Following the phosphorylation of tyrosines in the ITIM by Lyn, SH2-containing inositol 5′-phosphatase (SHIP) is recruited and phosphorylated. The SRC phosphatase, SHIP1/2, regulates cellular levels of PI(3,4,5)P3 by hydrolyzing it to form PI(3,4)P2, and this dephosphorylation inhibits cell proliferation. ITAM motifs do not always act to activate, but also to inhibit, transducing what is called an ITAM-mediated inhibitory signal (ITAMi). Unlike the ITIM, ITAMi does not require co-ligation with heterologous receptors to produce inhibitory signals. The bi-functionality of ITAMs is key to ensuring immune maintenance and to reduce the development of autoimmune diseases. ITAMi is activated following interaction with low affinity molecules such as FcαRI, FcγRIIA, and FcγRIIIA. Upon the binding of monomeric immunoglobulin to an FcR bearing the ITAM motif (e.g., FcγRIIA), the last tyrosine residue of the ITAM motif is phosphorylated by Lyn, which is responsible for the transient recruitment of Syk, followed by that of SHP-1, which halts the activation signal [27,28,29].

## 4. Biological Functions of FcγRs 

The FcγR family perform paradoxical functions as activators or inhibitors because of their structural heterogeneity, and are involved in regulating various immune responses. Along with their essential role in specific binding to the Fc portion of antibody subsets, FcγRs calibrate multiple effector responses, such as antibody-dependent cellular phagocytosis (ADCP), antibody-dependent cellular cytotoxicity (ADCC), release of inflammatory substances, B cell activation, DC maturation, antigen presentation, the release of inflammatory substances, and macrophage polarization [1,22,34,35,36].

Generally, Fc receptors perform three main functions. Firstly, they up- or down-regulate immune-cell responses, such as the proliferation of B cells, phagocytosis by macrophages, and the degranulation of mast cells, as well as the down modulation of immune responses. Secondly, they trigger the phagocytosis of captured immune complexes (ICs) after uptake, which leads to the eradication of the antigen–antibody complexes and the delivery of the antigenic peptides to the MHC class I or class II antigen presentation pathway [37]. Thirdly, they perform non-immunoregulatory functions; the neonatal FcR of IgG (FcRn) is responsible for the vertical transfer of maternal IgG, and the polymeric immunoglobulin receptor (poly-IgR) is responsible for the transfer of IgA to mucosal surfaces [38]. 

### 4.1. Roles of FcγRs in Innate Immunity

Owing to their prevalence on DCs and macrophages, FcRs are mediators for a wide range of afferent immune responses. For instance, the binding of immune complexes to activating FcRs on DCs and macrophages leads to phagocytosis, degradation, and the presentation of antigenic peptides via MHC class I and class II proteins and their ensuing recognition by T cell subsets [5].

Contrarily, the inhibitory FcγRIIBs retain the endocytosed antigens and expose them to B cells [39]. Additionally, the intracellular signaling capacity of FcγR directs inflammatory mediators, and the functional polarization and killing capability of macrophages and DCs [40]. Moreover, concomitant signaling of FcγR and TLR lowers IL-12 production and induces IL-10 and TNF release [41]. During the course of bacterial infection, *E. coli* induces an inhibitory FcRγ pathway by binding to CD16. This binding not only impairs macrophage receptor with collagenous structure (MARCO)-mediated bacterial clearance and phagocytosis but also activates tumor necrosis factor (TNF)-α secretion, which has an indirect impact on the exaggeration of the inflammatory response and thereby the spread of sepsis, as documented by enhanced survival and less pronounced sepsis in *FcRγ*^−/−^ mice compared to in wild type (WT) mice [42,43]. 

**Figure 1:**
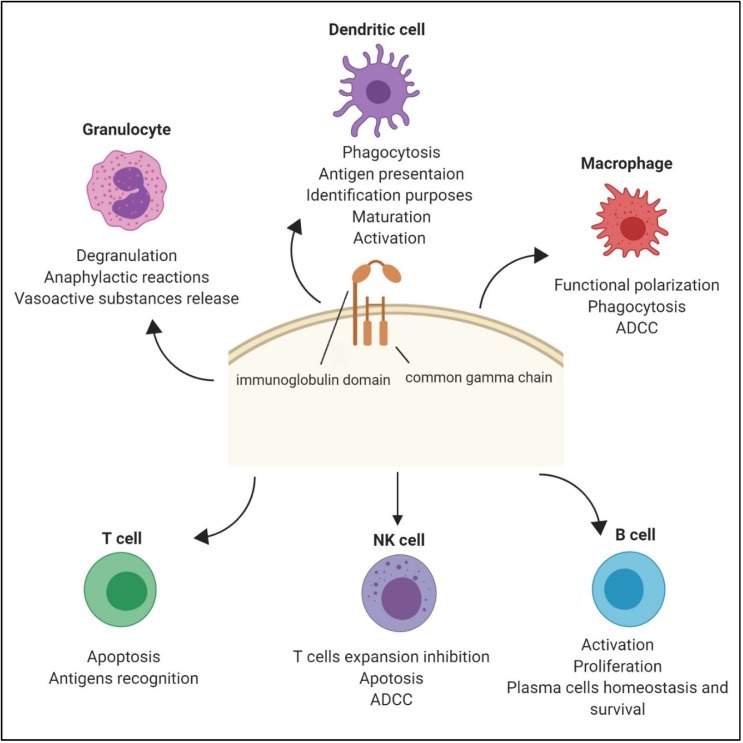
The biological functions of generic Fc gamma receptor (FcγR) (Created with BioRender.com).

Moreover, crosslinking of the immune complex to FcεRI—which is expressed on mast cells and basophils—results in basophil/mast cell degranulation and the release of vasoactive substances and chemoattractants, and thus leads to anaphylactic reactions [44]. In addition to their role in the activation of mast cells and basophils, FcεRIs are a mainstay in IgE-driven antigen presentation, as well as initiating the signaling pathways that incite allergic reactions [14,45]. By the same token, the high-affinity receptor FcγRI, which is expressed mainly on DCs but also on myeloid cells (including monocytes) and macrophages, binds monomeric IgG and can mediate ADCC and ADCP in response to crosslinking by antibodies [46]. In addition, OSCAR is broadly expressed on myeloid cells and is a receptor associated with FcRγ, which is a unique subunit on a variety of Fc receptors. OSCAR was reported to be necessary for antigen presentation and the activation of DCs in humans and mice [47]. In the context of fungal infections, Dectin-1—which is a C-type lectin pattern recognition receptor (PRR) and is expressed on macrophages, DCs and neutrophils—plays a key role in the response of DCs to the glucan components of the fungal cell wall [48]. The FcRγ subunit exerts inhibitory effects on Dectin-1 signaling in DCs [49]. Furthermore, the C-Type Lectin Receptor (CLR) DC immunoreceptor (DCIR) is important for the development of cerebral malaria [50]. Similarly, FcRγ and DAP 12 have inhibitory roles in the TLR response in macrophages [51]. Furthermore, DCs and macrophage-intrinsic Dectin-1 are indispensable for NK cell-dependent tumor cell eradication [52]. Besides its functional role in DCs, FcγR has an intrinsic role in helping the phenotypical characterization of DC. For example, FcγR could be employed as an identification marker to differentiate between monocyte-derived dendritic cells (moDCs), macrophages from conventional DCs (cDCs), and plasmacytoid DCs (pDCs), being more highly expressed on the first (Figure 1) [53,54,55].

### 4.2. Roles of FcγRs in Adaptive Immunity

On human and murine B cells, the only Fc receptor expressed is the inhibitory FcγRIIB, which is of prime importance for maintaining antibody-mediated immunity through negative effects on B cell functions and regulating plasma cell homeostasis and survival [1,31]. 

With the exception of NK cells and T cells, the ubiquitous inhibitory low-affinity FcR for IgG, FcγRIIB—which is co-expressed with other aforementioned activating receptors on nearly all leukocytes—is present in two forms: FcγRIIB-1, which is expressed solely on B cells, and FcγRIIB-2, which is expressed on all other cell types and can efficiently control antibody-mediated responses [1]. NK cells solely express the activating receptor FcγRIII (CD16)—at high levels in humans, and modest levels in mice [56]—which consists of both FcγRIIIα and FcRγ chains, and plays a paramount function in IgG-dependent cell cytotoxicity and the production of many cytokines and chemokines. It was found that NK cells can be activated with the aid of IgE via binding to FcγRIII, with ensuing cytokine production and ADCC, culminating in IgE-mediated allergic reactions [57]. Furthermore, it was reported that disintegrin and metalloprotease-17 (ADAM17) influence CD16 phenotypically and functionally [58]. Moreover, monocytes enhance ADCC mediated by NK cells by means of FcγRII (CD32) [46,59].

Disparately, the FcRγ subunit plays a novel role in promoting T cell apoptosis along with Fas-Fas ligand and through the activation of caspases 3 and 9 [60]. For example, murine FcγRII (CD32) contributes to apoptosis in granulocyte precursors and in degranulated eosinophils via the Fas-Fas ligand mechanism [61]. Similarly, FcγRIII (CD16) promotes the apoptosis of NK cells [62]. Moreover, an earlier study found that early fetal thymocytes expressing FcγRII/III+ and double negative for CD4 and CD8 host precursors for T lymphocytes and NK cells [63]. Even though T cells do not express FcRs, a very recent study has shown that the FcRγ subunit hampers virus-specific cytotoxic T cells in an NCR1-dependent manner (Figure 1) [11].

### 4.3. Roles of FcγRs in Non-Immune Cells

FcγR functions are not restricted to immune cells. For instance, non-immune cells express the FcRγ subunit, such as with glycoprotein VI (GPVI). GPVI, existing as a complex of two GPVI molecules and one FcRγ-chain dimer complex, is the main activation receptor on platelets for collagen, by which collagen mediates the activation and aggregation of platelets [14,64]. Furthermore, FcγRI on neurons binds to IgG and assists in the production of neurotransmitters [65]. Besides their role in normal neurodevelopment, neuronal FcγRs in the brain have been found to mediate brain neurocytotoxicity via kainic-acid- and amyloid-β-dependent mechanisms [66]. Microglia, the innate immune cells of the brain, are known to express FcγR, which mediates microglial phagocytosis of traumatized cells [66]. Liver sinusoidal endothelial cells (LSECs) play a key role in controlling the exchange of molecules between the blood and hepatocytes [67], and they remove waste materials from the blood through the expression of scavenger receptors [68]. Low-affinity gamma immunoglobulin Fc region receptor IIb (FcγRIIb) is among the immunoreceptors expressed on LSECs that mediate the endocytosis of waste materials [69]. Pathologically, it was found that FcγRIIb on LSECs was lost in most human and murine hepatocellular carcinomas [70]. Fibrinogen-like protein 2 (FGL2), a ligand for FcγRIIb [71], was reported to be increased in patients with non-alcoholic fatty liver disease (NAFLD) [72] and liver cirrhosis [73]. In another study, FcγRIIb on liver endothelial sinusoidal cells was found to eliminate immune complexes [74]. Furthermore, the endothelial cell-intrinsic FcγRIIb was observed to be linked with hepatic [75] and cardiovascular diseases [76].

Unconventionally, various bacteria and parasites express Fc receptors; such pathogens include schistosomes, trypanosomes, staphylococci, pneumococci, and streptococci [7]. Intriguingly, host cells virally infected with HCMV, HSV, EBV, and VZV express specific virus-encoded Fc receptors that bind the Fc portion of IgG, acting as inhibitors of IgG-mediated immunity [77,78]. In the context of persistent chronic virus infections such as LCMV, it was found that FcγR-dependent, Ab-mediated effector functions that guarantee the killing of infected cells and antigen presentation of T cells were impaired due to high amounts of immune complexes [79,80].

## 5. Therapeutic Approaches Using FcRs

FcRs are the mainstay regulators of the immune system by virtue of their ability to calibrate tolerance and prime the effector functions of humoral and cellular components. Indeed, targeting these adaptor proteins shows promise for impact in immunotherapeutic settings. Compelling evidence from several studies emphasizes the importance of inhibitory FcγRIIB in maintaining peripheral tolerance [81] and humoral tolerance [82]. In the case of an imbalance between activating and inhibitory FcRs, a state of IC-triggered autoimmune disease ensues; the activation of FcRs primes the autoimmune pathology, and the functional restoration of inhibitory FcγRIIB in humans and mice by an agonist ameliorates the disease [83]. Additionally, downmodulation or neutralization of the FcγR interactions with immune complexes via an antagonist precludes the IC-mediated inflammatory responses that are driven by autoantibodies [84]. The reverse scenario is also plausible; amplifying the activity of antibodies by enhancing their interaction with activating FcγRs—which are mediators of ADCC—or blocking the binding of antibodies to the inhibitory receptor, and hence amplifying ADCC reactions is promising in immunotherapeutic approaches. Furthermore, FcRs may be targeted either with variant antibodies to facilitate the relay of antigens into DCs for antigen presentation, or the alteration of the Fc subunit of therapeutic antibodies at the post-translational level [1,21,38].

## 6. Concluding Remarks

As previously mentioned, FcRs have a substantial impact in shaping not only the afferent and efferent immune responses, but also non-hematopoietic cell biological functions, by virtue of their structural diversity and functional heterogeneity. Delineating the biology of FcRs and their unique constituent, FcεRIγ, is of importance to unravel antibody effector functions, and to establish therapeutics tools in alloreactivity or cancer settings. 

## Figures and Tables

**Table 1:** Summary of human and murine Fc receptor biology. This table was compiled using information from [19,27,30,31,32,33].

Name	Alternative Name (CD Marker)	Structure	Gene	Cellular Avenues	Affinity	Classification	Function
FcγRI/FcγRIA	CD64/CD64a	αγγ	*fcgr1/FCGR1A*	Macrophages, Neutrophils, Eosinophils and dendritic cells	High/IgG/mouse and human	Canonical (type I)	activating
FcγRIIb	CD32b	α	*fcgr2b/FCGR2B*	Macrophages, Neutrophils, basophiles, dendritic cells and B cells	Low to medium/IgG/mouse and human	Canonical (type I)	inhibitory
FcγRIIa	CD32a	α	*FCGR2A*	Monocytes, Neutrophils and NK cells	Low to medium/IgG/human	Canonical (type I)	activating
FcγRIIc	CD32c	α	*FCGR2C*	Macrophages, Neutrophils, dendritic cells, mast cells, eosinophils and platelets	Low to medium/IgG/human	Canonical (type I)	activating
FcγRIII	CD16	αγγ	*fcgr3*	Macrophages, Neutrophils, dendritic cells and NK cells	Low to medium/IgG/mouse	Canonical (type I)	activating
FcγRIIIa	CD16a	αγγ	*FCGR3A*	Macrophages and NK cells	Low to medium/IgG/human	Canonical (type I)	activating
FcγRIIIb	CD16b	α-GPI	*FCGR3B*	Neutrophils and basophils	Low to medium/IgG/human	Canonical (type I)	activating
FcγRIV	-------	αγγ	*fcgr4*	Macrophages, Neutrophils and dendritic cells	Low to medium/IgG/mouse	Canonical (type I)	activating
FcαRI	CD89	αγγ	*fcar/FCAR*	Macrophages, neutrophils and Eosinophils	Low/IgA/mouse and human	Canonical (type I)	activating
Fcα/µR	CD351	Ig-like domain	*fcamr/FCAMR*	B cells, macrophages, and activated T cells	High/IgA and IgM/mouse and human	Canonical (type I)	No canonical ITAM/ITIM domains
FcεRI	-------	αβγγ	*fcer1g/FCER1G*	Mast cells, Basophiles, monocytes and Langerhans	High/IgE/mouse and human	Canonical (type I)	activating
FcεRII	CD23	C-type lectin-like domain	*fcer2/FCER2*	B cells, T cells, Monocytes, Macrophages and Eosinophils	Low/IgE/mouse and human	Non-canonical (C-type lectin)	activating
CD22	--------	SIGLEC family of lectins	*CD22*	B cells	sialiated IgG/mouse	Non-canonical (C-type lectin)	inhibitory
FcRn	---------	Similar to MHC-I and associates with β2m	*fcgrt/FCGRT*	Endothelial and Epithelial cells	High/mouse and human	Non-canonical (MHC-I)	No canonical ITAM/ITIM domains
SIGNR1	CD209b	β2m	*CD209*	Macrophages and dendritic cells	sialiated IgG/mouse	Non-canonical (C-type lectin)	No canonical ITAM/ITIM domains
DC-SIGN	CD209	β2m	*CD209*	dendritic cells	sialiated IgG/human	Non-canonical (C-type lectin)	No canonical ITAM/ITIM domains
DCIR	CD367	C-Type Lectin-Like Receptor	*clec4a*	DCs, but also by macrophages, monocytes, and B cells	sialiated IgG/mouse	Non-canonical (C-type lectin)	inhibitory

**Abbreviations:** CD, cluster of differentiation; FcR, fragment crystalizable receptor; FcRn, neonatal Fc receptor; SIGNR1, specific ICAM-3 grabbing nonintegrin-related 1; DC-SIGN, dendritic cell-specific intercellular adhesion molecule-3-grabbing non-integrin; DCIR, CLR (C-type lectin receptors) dendritic cell immunoreceptor; clec4a, C-type lectin domain family 4 member A; GPI, glycosylphosphatidylinositol; Ig, immunoglobulin; MHC, major histocompatibility complex.
